# Secure Attachment Priming Amplifies Approach Motivation for Infant Faces Among Childless Adults

**DOI:** 10.3389/fpsyg.2021.736379

**Published:** 2021-10-27

**Authors:** Fangyuan Ding, Yuncheng Jia, Gang Cheng, Lili Wu, Tianqiang Hu, Dajun Zhang

**Affiliations:** ^1^Faculty of Psychology, Southwest University, Chongqing, China; ^2^Center for Mental Health Education, Southwest University, Chongqing, China; ^3^School of Psychology, Guizhou Normal University, Guiyang, China; ^4^Center for Rural Children and Adolescents Mental Health Education, Guizhou Normal University, Guiyang, China; ^5^Brain and Cognitive Neuroscience Research Center, Liaoning Normal University, Dalian, China

**Keywords:** security priming, infant faces, liking, wanting, motivational responses, kindchenschema

## Abstract

Existing studies have indicated that priming secure attachment alters adults’ neural responses to infant faces. However, no study has examined whether this effect exists for motivational behavioral responses, and none of the previous studies included adult faces as a baseline to determine whether the security prime enhances responses to human faces in general or infant faces alone. To address this limitation, the current study recruited 160 unmarried and childless adults in the first phase, and all of them completed a battery of questionnaires, including the Interest in Infants, the Experiences in Close Relationships (ECR), and State Adult Attachment Measure (SAAM). A week later, after priming, 152 (76 security-primed vs. 76 neutrally primed) participants completed the SAAM and a behavioral program assessing their motivational responses to both adult and infant faces (i.e., liking, representational, and evoked responses). A manipulation check showed that the security prime was effective. Then, generalized linear mixed-effects models (GLMMs) showed that security priming enhances adults’ liking, representational, and evoked responses (three components of the motivational system) only to infant faces and not to adult faces. Moreover, hierarchical regression analysis indicated that, even after security priming, there was a substantial linear relationship between positive motivation toward infant faces and the state of adult secure attachment. In summary, this study demonstrated for the first time that promoting the state of adult secure attachment can effectively enhance the effect size of the baby face schema. The current results were interpreted according to Bowlby’s view of the attachment behavioral system.

## Introduction

Kindchenschema (or baby schema) is an important concept proposed by Lorenz (1943) that refers to a series of appearance features typical of young animals or human babies that can cause adults to perceive these infants as cute, motivating their concern and caretaking behavior (Parsons et al., 2010). As a kind of innate release mechanism, the kindchenschema can widespread arouse a series of positive responses in adults, which is considered to be the basis of human nurturing behavior and the construction of early parent-child relationships (Kringelbach et al., 2016; Franklin and Volk, 2018; Kou et al., 2020).

Since the concept of kindchenschema was proposed, many researchers have studied it and found that adults can effectively evoke the sense of “cuteness” when facing infants’ appearance, odor, sound, and other related cues (Kringelbach et al., 2016; Kou et al., 2020). Among these cues, the face of the infant is thought to be the most typical representative region of the kindchenschema (Glocker et al., 2009). Compared with adult faces, infant faces have larger eyes, which are located in the middle of the face. In addition, infants have a large forehead and relatively small nose, mouth, and chin. These facial features comprise the baby face schema (Rayson et al., 2017). Hence, follow-up studies on kindchenschema mainly focus on baby face schema (Rayson et al., 2017).

At present, numerous studies have found that the infant face provides hedonic stimulation for human beings [see reviews in Kringelbach et al. (2016) and Kou et al. (2020)] and can evoke positive attitudes and experiences related to parenting [see review in Franklin and Volk (2018)]. For example, empirical studies have found that in adults, infant faces can effectively evoke and induce rapid cognitive and behavioral responses (Endendijk et al., 2018), positive emotional responses (Almanza-Sepúlveda et al., 2018), and a strong inclination to protect and care for infants (Cheng et al., 2015b). Generally, adults have an obvious preference for infant faces.

Although the preference for infant faces is considered to be an innate release mechanism, it is influenced by some acquired factors (for review, see Luo et al., 2015). Among them, attachment is an internalized representation of early experiences that serves as a template for people’s thoughts, feelings, and behaviors throughout their life (Bowlby, 1969/1982; Johnson et al., 2010). At present, a large number of studies on attachment and parenting have found that secure attachment is evidently related to the cognition, emotion, and behavior of parenting, while the opposite has been found for insecure attachment (Jones et al., 2015a, b; Shlafer et al., 2015; Negrao et al., 2016; Olsavsky et al., 2020).

Regarding the preference for infant faces, relevant cognitive neuroscience research has found that when viewing infant faces, adults’ neural activity related to maternal behaviors is affected by their own attachment (Strathearn et al., 2009; Lenzi et al., 2013; Groh and Haydon, 2017). Specifically, secure mothers exhibited greater activity in the ventral striatum and hypothalamus/pituitary, and these brain regions are considered to be associated with reward. In contrast, mothers with insecure/dismissive attachment showed increased anterior insula activity when they looked at the sad faces of their own baby, which is considered to be associated with negative feelings such as disgust, pain, and unfairness (Strathearn et al., 2009). Another study of nulliparous females found that secure females had more activation in the medial orbitofrontal cortex and pregenual anterior cingulate cortex (ACC) when empathizing with infant faces, while avoidant females exhibited the reverse (Lenzi et al., 2013). Furthermore, in one fMRI study on childless adults, whole brain regression analysis showed that the activation in the insula and inferior orbitofrontal cortex to infant crying faces was positively related to state secure attachment, whereas enhanced cuneus activity to infant joy was correlated to anxious attachment state (Ma et al., 2020). Through ROI analysis, this study also found that increased activation in the insula and ACC to infant crying were positively related to attachment anxiety state, while a higher attachment avoidance state was correlated with decreased amygdala activity to infants and attenuated insula and ACC activation to infant joy. In addition to these fMRI studies, ERP studies found that insecure mothers had more difficulties processing infant faces with negative emotion (higher N170 amplitude), and more attention resources were allocated to infant faces (increased P3 amplitude) by secure mothers (Fraedrich et al., 2010; Leyh et al., 2016). Similar findings were also found among nulliparous females (Ma et al., 2017).

The above studies preliminarily proved that individuals with secure attachment are generally more sensitive to infant faces. Recently, further research using attachment priming techniques found that alteration of the attachment state can significantly change adults’ brain responses to infant faces (Ma et al., 2019a, b). Specifically, priming security supraliminally or subliminally by presenting images that depict people enjoying caregiving behaviors and intimate attachment relationships could uniformly enhance early selective attention (larger N1, shorter P2 latencies, or larger P2) and later controlled attention (larger P3) to infant faces in insecure women (Ma et al., 2019b). Under insecurity primes, by presenting pictures of negative parent-child attachment scenes, the N1 and P2 amplitudes of secure women were significantly increased, which means that secure women allocated more attention resources to infant faces; in contrast, the N1 and P3 amplitudes of insecure women were significantly suppressed (Ma et al., 2019a).

In summary, attachment can significantly affect adults’ neural responses to infant faces, and there is an obvious covariant relationship between these neural responses and attachment state. However, there are still some problems to be solved. First, as Endendijk et al. (2018) pointed out, there is a distinction between the baby face schema effect and the response to the baby face. If researchers want to strictly extract the exclusive effect of the baby face schema, they should use the following two ways to study: (1) taking adult faces as the baseline, the response differences between infant and adult faces were compared to obtain the effect of the baby face schema and (2) manipulating the typical features of the baby face schema in infant faces to extract the exclusive effect of the baby face schema. In fact, previous studies have found that human faces serve as a special stimulus in which individuals with different attachment orientations show different responses (Dan and Raz, 2012; Kafetsios et al., 2014; Zheng et al., 2015; Tang et al., 2017; Petrowski et al., 2019; Alaei et al., 2020). However, the above studies simply investigated the relationship between attachment and the response to infant faces (Strathearn et al., 2009; Lenzi et al., 2013; Groh and Haydon, 2017; Ma et al., 2019b, 2020). Therefore, it remains unclear whether the results of these studies reflect the common characteristics of the response to human faces or a unique response to baby faces. Second, most of the existing studies investigated only differences in the neural processing of baby faces among individuals with different attachment orientations. Therefore, whether these differences exist in motivational behavioral responses is also a question worth exploring.

To address these shortcomings, Cheng et al. (2015b) developed a motivation response test program based on Heerey and Gold (2007) behavior research paradigm. The task uses pictures of adult and infant faces as stimulus material to elicit wanting and liking responses and includes three indicators: (1) Liking, which refers to the hedonic value of each stimulus and is obtained by self-report of participants’ hedonic experience when viewing the face pictures; (2) Representational response, which refers to the degree of effort exerted to seek or avoid future exposure of face pictures, and the memory representation of face pictures is required to be measured by the key-press procedure after the face pictures disappear; and (3) Evoked response, which refers to motivated behavior in the presence of face pictures and is obtained by a key-press procedure to examine how hard participants will work to prolong or decrease exposure to the current perceptible face pictures. According to the definition of Berridge and Robinson (2003), wanting refers to the motivation to make a series of efforts to obtain a desirable reward; liking refers to the degree of pleasure that participants feel when they consume reward stimulation. Therefore, in the behavioral tasks used by Cheng et al. (2015b), Liking and Representational response represent liking and wanting, respectively, as proposed by Berridge and Robinson (2003), while the Evoked response is a mixture of the two. In addition, the test results obtained in this behavior paradigm have been proven to be related to brain circuitry processing rewarding stimuli (Aharon et al., 2001).

Cheng et al. (2015b) also combined the motivation response test program with a verbal test to investigate the associations between state attachment and baby face schema in a group of childless adults. After controlling for gender, state avoidant attachment, and state anxious attachment, individuals with higher state secure attachment showed higher interest in infants under verbal test and stronger representational and evoked responses and self-reported higher liking to neutral infant faces. This means that the baby face schema effect becomes stronger as the level of secure attachment increases. Subsequently, Ding et al. (2016) used infant facial expression pictures to confirm this result.

To some extent, these two research results demonstrate that consistent with previous studies that discovered diverse neural activities when watching infants faces individuals with different levels of secure attachment (e.g., Strathearn et al., 2009; Lenzi et al., 2013; Groh and Haydon, 2017; Ma et al., 2019b, 2020), motivational behavioral responses differ by level of attachment security (Cheng et al., 2015b; Ding et al., 2016). However, it is still unknown whether the baby face schema effect changes with changes in attachment state, given that both studies were cross-sectional. Moreover, although there have been existing studies showing that attachment priming can change the neural response of adults to images of infant faces (Ma et al., 2019a, b), this change in attachment state may also lead to similar changes in subjects’ responses to adult faces (Tang et al., 2017). In other words, changes in attachment state may lead to changes in the response of subjects to both adult and infant faces at the same time and thus no changes in the size of the baby face schema effect.

To address this limitation, the current study employed a 2 (between-subject factor: experimental group, control group) × 2 (within-subject factor: baby faces, adult faces) mixed design. Specifically, the subjects in the experimental group received explicit priming of attachment figure representations to enhance the subjects’ state of secure attachment, and the subjects in the control group received neutral priming (i.e., recalled a school acquaintance). Then, all subjects completed a behavioral task that measured the motivational responses to both adult and infant faces. We aimed to investigate whether the hypothesized increase in the state of secure attachment alters the baby face schema effect. Moreover, although security priming would enhance the state of secure attachment, there would still be significant individual differences in the subject’s attachment state. Thus, we also planned to perform regression analysis similar to the study of Cheng et al. (2015b) to see whether there are linear relationships between the scores of secure attachment state after priming and the scores of unique motivational responses to infant faces (subtracting responses to adult faces from responses to infant faces). The specific research hypotheses are as follows:

Hypothesis 1: There would be significant main effects of group on motivational responses. Specifically, the motivational responses to infants’ and adults’ faces in the experimental group were higher than those in the control group.

Hypothesis 2: There would be significant interactive effects between group and face type on motivational responses. Specifically, compared with the control group, the experimental group had more motivational responses to infant faces than to adult faces.

Hypothesis 3: After priming, the state of secure attachment of the two groups will still effectively predict unique motivational responses to infant faces.

## Materials and Methods

### Participants

This study was carried out in two phases. First, we recruited a sample similar to previous studies (Cheng et al., 2015b; Ding et al., 2016) to ensure comparability. Specifically, 160 unmarried and childless undergraduates (80 men, 80 women), ranging in age from 18 to 25 years old (*M*_*age*_ = 20.51, SD = 1.47), were recruited from our university. All subjects were assigned to either the experimental group (40 males, 40 females) or the control group (40 males, 40 females) according to their data collected in this phase.

Due to personal reasons, some subjects dropped out of the study in the second phase. Thus, the sample in the second phase consisted of the experimental group, with 76 participants (37 men, 39 women), aged 18–24 years (*M* = 20.43, SD = 1.38), and the control group, with 76 participants (39 men, 37 women), aged 18–25 years (*M* = 20.61, SD = 1.58). No significant difference between the groups by gender (*χ*^2^ = 0.105, *df* = 1, *p* = 0.746) or age (*t*_(__150__)_ = −0.712, *p* = 0.477, Cohen’s *d* = −0.121) was found. Twenty yuan were compensated for participants’ involvement. The Ethics Committee of the first author’s university approved this study (No. 2014179).

### Measures

#### The State Adult Attachment Measure

The State Adult Attachment Measure (SAAM), developed by Gillath et al. (2009), is a 21-item self-report measure capturing temporary fluctuations with respect to attachment. The Chinese version of SAAM (Ma et al., 2012) was used. The scale has three dimensions: security, anxiety, and avoidance and is rated from 1 (strongly disagree) to 7 (strongly agree). The psychometric data ranges of the three subscales are security, 9–63; avoidance, 7–49; and anxiety, 5–35. The Cronbach’s α values in the present study were 0.73 for security, 0.69 for avoidance, and 0.70 for anxiety.

#### Experiences in Close Relationships

The Experiences in Close Relationships (ECR; Brennan et al., 1998), the most common scale of attachment patterns in adults, consists of 36 items in total with 18 items each assessing attachment avoidance and anxiety. Participants rated the items on a 7-point Likert scale with 1, 4, and 7 representing strongly disagree, neutral/mixed, and strongly agree, respectively. The current study used the Chinese version (Li and Kato, 2006), which has proven to be a reliable scale with good psychometric properties. The Cronbach’s α values in the current sample for the total questionnaire and the subscales measuring avoidance attachment orientation and anxiety attachment orientation were 0.82, 0.80, and 0.85, respectively.

#### Interest in Infants

The present study employed the 10-item revised Interest in Infants (Maestripieri and Pelka, 2002; Charles et al., 2013), which was translated by Cheng et al. (2015b) and has excellent psychometric properties. The questionnaire is based on the following primary question: “*If you were at a party and there was a baby in the room that you did not know, what would you most likely do?*” Then, participants reported their likelihood of engaging in ten different types of listed activities (e.g., “*Reach out and touch the baby or smile and talk to it*”) on a 6-point scale, ranging from 1 (not at all likely) to 6 (very likely). Two reverse-coded items are about avoidance of the infant. We summed the scores for all items, with high scores representing a high interest in infants. In the current sample, the Cronbach’s α value is 0.87.

#### Computerized Display

**FIGURE 1:**
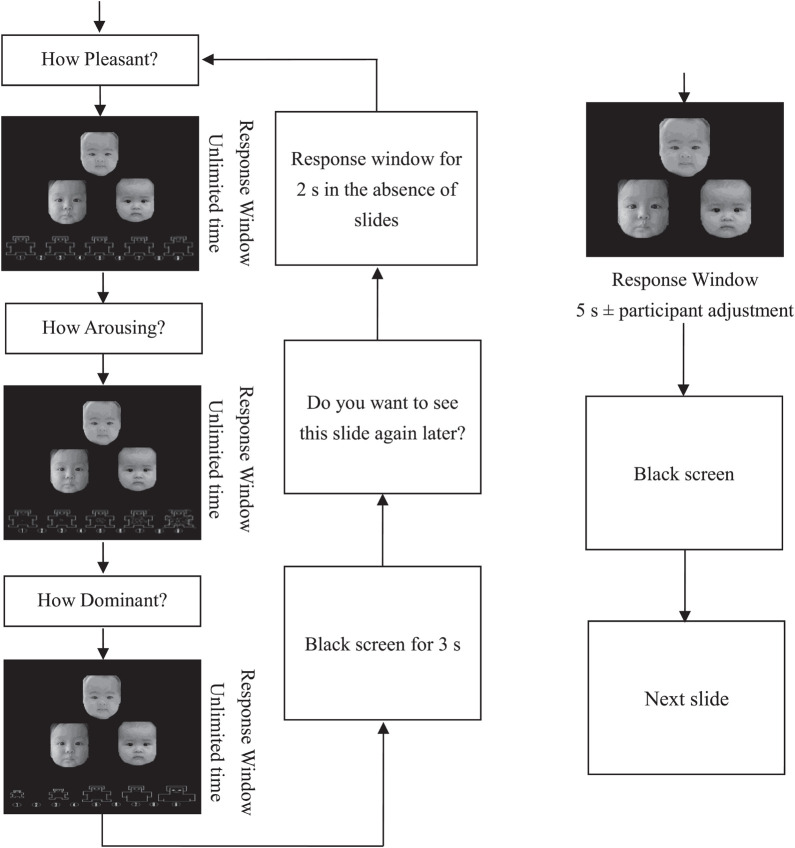
Experimental procedure. The first block contains part A and part B, which are presented alternately. After the completion of the first block, part C commences. These infant faces were reprinted from the Chinese Infant Affective Face Picture System (CIAFS) under a CC BY license, with permission from GC, original copyright (2015).

The motivation response test program involves the use of a computer to evaluate the motivation of participants with regard to viewing unfamiliar infant faces and was used in prior studies by Cheng et al. (2015b) and Ding et al. (2016). The program consists of three parts, which simultaneously measure liking, representational response, and evoked response in a watching motivation system (see Figure 1). It uses E-prime 2.0 for programming and testing.

Part A is mainly used to measure the liking component of the watching motivation system. In this part, the participants were asked to view and evaluate 16 slides, each made up of three different neutral face pictures. Of these slides, 8 slides were composed of infant face pictures, and the other 8 slides (half male and half female) were composed of same-gender face pictures of adults. These pictures are from the Chinese Infant Affective Face Picture System (Cheng et al., 2015a) and the Chinese Affective Face Picture System (Gong et al., 2011). The expression intensity of the three faces in each slide was similar, and there was no statistically significant difference between the infant and adult faces in intensity (*t*_(__46__)_ = 0.75, *p* = 0.46, Cohen’s *d* = 0.218). In this part, participants were instructed to view the slides and report their experience as pleasant, dominant, or arousing *via* the Self-Assessment Manikin (Lang et al., 1999); each of the slides was rated on a 9-point Likert scale (1 = extremely calming/unpleasant/dominant; 9 = extremely arousing/pleasant/dominant). The average score of participants for the infant or adult faces was calculated for pleasure, arousal, and dominance. Among these ratings, only the pleasure rating was considered to reflect the liking component of the motivation system. Arousal and dominance ratings were also collected in this study, merely to ensure that the program was identical to the one used in the previous cross-sectional study (Cheng et al., 2015b). However, given that the ratings of arousal and dominance were irrelevant to the purposes of this study, the results of these two indicators are not presented in the main paper; interested readers can find them in the Supplementary Material.

Part B was completed after the assessment of each slide and was used to test representational response. At the beginning of the experimental program, participants were informed that some of the slides they had evaluated would be viewed again in a later part of the test. They were also told what slides they would see next, depending on what they did in this section. For the slides that they were willing to see again, participants could increase the probability of their reoccurrence by rapidly pressing the “n” and “m” keys alternately. In contrast, for the slides they did not want to see again, participants could decrease the probability of their reoccurrence by rapidly pressing the “x” and “z” keys alternately. During this part of the testing process, we also asked participants to press keys only for slides that they do not want/want to see again and to not respond to slides that they do not matter.

Part C was used to measure the evoked response of participants, which is understood as the motivational behavior under exposure to stimuli. In this section, participants were asked to view 12 slides (6 infants, 3 adult males, and 3 adult females) that had been seen before. In fact, all participants saw the same slides. While participants were viewing these slides, they could control the viewing time by pressing certain keys. For the slides they wanted to see, participants could extend the presentation time by rapidly pressing the “n” and “m” keys alternately; conversely, participants could rapidly press the “x” and “z” keys alternately to shorten the presentation time for each slide. If participants did not press any keys, each slide appeared for 5 s. In addition, the maximum presentation time a participant could view any slide was 10 s. Like part B, participants could choose not to press any keys if they did not care how long the slide was presented, and they were informed that the total time to complete the task would not be altered by their key presses.

Representational (the wanting component of the motivation system) and evoked responses (the mixture of liking and wanting) were derived by subtracting the total number of presses made to seek desirable slides within one type of face from the total number of presses made to avoid undesirable slides within one type of face. In this way, the net interest value for infant or adult faces was obtained for each subject. In other words, representational responses for each face were measured by the net total keystrokes to seek to see each face again with the absence of stimuli, and evoked responses for each face were measured by the net total keystrokes to prolong the viewing time of each face.

### Procedure

In the first phase, 160 participants were taken to a classroom where the questionnaire was administered in a group setting. Before the experiment began, the researchers first provided preliminary information about the study. Then, after the participants signed the informed consent form, the battery of questionnaires was distributed and administered; it contained a demographic information survey and a battery of questionnaires, including the SAAM, the ECR, and the Interest in Infants. Based on the results of this phase, the subjects were paired according to their gender and their scores on the SAAM. The experimental group and the control group consisted of 80 participants split equally by gender.

Next, 7 days after the completion of the questionnaires, the subjects were tested in the second phase. This phase involved three steps. The first step was an attachment priming procedure, which was based on the materials used in Gillath et al. (2009) and has been proven effective for improving participants’ state of secure attachment (Pan et al., 2016). The participants in the experimental group (security priming, 76 participants) were asked to recall someone close or supportive to them and then describe the face and several traits of this person and the time when he or she offered help (security priming) in a paper-and-pencil questionnaire. In the control group (neutral priming), 76 participants were asked to recall a school acquaintance and write down a time when they study together. The participants then completed the computer task. Finally, the SAAM questionnaire was completed by the participants again to validate the effect of their attachment priming. To control the expected effect of the experimenter, a double-blind design was used in this phase. The whole process was approximately 40–45 min in duration. At the end of the experiment, participants were debriefed in detail.

### Data Analysis Strategies

All data analysis was performed using SPSS 26 in the current study. First, Pearson correlational analysis, regression analysis and independent *T*-tests were performed on the data collected in the first phase to describe the characteristics of the two groups before priming. Second, to verify the effectiveness of secure attachment priming, 2 [group (between-subject factor): security priming vs. neutral priming] × 2 [time (within-subject factor): prepriming vs. postpriming] ANOVAs were conducted to test the three attachment states of the two groups.

Third, generalized linear mixed-effects models (GLMMs) were used to test hypothesis 1 and 2. The advantage of a GLMM over repeated-measures ANOVA is that it can consider individual random variances and stimulus variation while examining fixed effects. In this study, the data structure was defined as slides nested within face types, face types nested within subjects, and subjects nested within groups. According to the recommendations of Matuschek et al. (2017), we employed “a minimal to maximal-that-improves-fit process” to select random effects. Since the effect of interest in this study is the fixed interaction effect between group (between-subject factor) and face type (within-subject factor), the minimal GLMMs included all fixed effects (the interaction effect and main effects of group and face type) and two random effects (i.e., random intercepts for subject and slide), and the maximal GLMMs included all fixed effects and three random effects (two random intercepts and random slopes for face type). Both group and face type were dummy coded (1 = experimental group/infant face, 0 = control group/adult face). The models were estimated with the restricted maximum likelihood method, and the Kenward–Roger approximate degrees of freedom were used to test the significance of the coefficients of fixed effects. Different repeated covariance types for both minimal and maximal GLMMs were also considered.

Then, the Akaike information criterion (AIC) and Schwarz’s Bayesian information criterion (BIC) were used to compare non-nested models, with smaller values indicating better model fits. Moreover, the likelihood ratio test (LRT) was used to compare nested models, and if the maximal LMM did not significantly improve the model fit, the minimal LMM was chosen. The full process for GLMM estimation is provided in the Supplementary Excel Sheet, and we report only the results of the final selected models in the results section.

Finally, similar to the study of Cheng et al. (2015b), we calculated the unique motivational response to infants using the response to adult faces as a baseline. Specifically, we used participants’ responses to the infant faces minus their response to the adult faces to calculate the specific scores for liking, arousal, dominance, representational and evoked responses for the infant faces. Then, hierarchical regression analyses were performed to examine the role of secure attachment state after priming in unique motivational responses as well as arousal and dominance ratings to infant faces. To exclude the confounding effects of gender and anxious and avoidant attachment states, these variables were entered into the regression models first (step 1). Next, a secure attachment state was entered (step 2), and the changes in *R*^2^ that secure attachment state brings were reported.

## Results

### Characteristics of Two Groups Before Priming

To investigate whether the participants recruited in this study were consistent with those in the study by Cheng et al. (2015b), we first conducted a correlation analysis (see Table 1) and regression analysis of the results from the first phase. The results showed that secure attachment (*β* = 0.198, *p* = 0.027) positively predicted the Interest in Infants score after we controlled for gender (*β* = 0.277, *p* < 0.001) and the states of anxious attachment (*β* = 0.126, *p* = 0.147) and avoidant attachment (*β* = 0.041, *p* = 0.589).

**TABLE 1:** Descriptive statistics and correlations (*n* = 160).

	1	2	3	4
1. Secure attachment	–			
2. Anxious attachment	0.495***	–		
3. Avoidant attachment	–0.132	0.081	–	
4. Interest in infants	0.313***	0.220**	0.054	–
5. Gender	0.209**	–0.024	0.105	0.320***
*M*	46.919	22.131	22.637	42.031
*SD*	7.819	5.393	7.381	9.750

**p < 0.01, ***p < 0.001.

*Gender was dummy coded (male = 0, female = 1).*

For personal reasons, some subjects did not participate in the second phase of the study. Finally, a total of 152 participants completed the test, namely, an experimental group of 76 participants (37 men, 39 women) and a control group of 76 participants (39 men, 37 women). Independent samples *T*-test results showed that the experimental group was not significantly different from the control group in the SAAM, ECR, and Interest in Infants scores (*t* = 0.151∼1.076, *p* = 0.284∼0.880). This indicates that the two groups of participants were homogeneous with respect to the relevant variables before attachment priming.

### Manipulation Check

**FIGURE 2:**
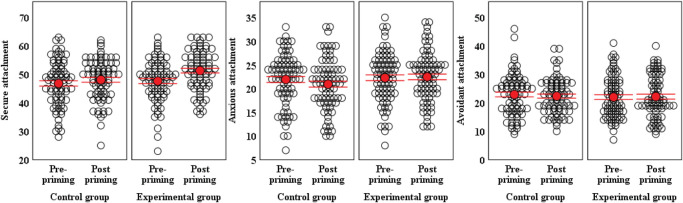
Univariate scatterplots of the attachment state at pre- and postpriming in each group. The error bars represent ± 1 standard error (red line) of the mean (red point).

According to the results presented in Table 2, the main effects of group and time and the interactive effect of group and time on anxious and avoidant attachment were not significant, whereas for secure attachment state, the main effect of time and the interactive effect were significant and the main effect of group were close to significant. The results of Bonferroni-corrected *post hoc* multiple comparisons showed that after attachment priming, the experimental group showed a significantly higher state of secure attachment than the control group (*p* = 0.006) and no differences between the two groups before priming (*p* = 0.506). Moreover, for the experimental group, the secure attachment state after priming was significantly higher than that before priming (*p* < 0.001), whereas for the control group, the differences between the state of secure attachment before priming and after priming were not significant (*p* = 0.123). This indicates that attachment priming was effective in this study and successfully improved the level of secure attachment in the experimental group. Figure 2 was plotted to make the above results more intuitive.

**TABLE 2:** Analysis of the priming effect of the attachment state (*n* = 152).

		Time (*M* ± *SD*)		Group			Time			Group × Time		
Dependent variable	Group	Prepriming	Postpriming	*F (df1,df2)*	*p*	$\eta_{p}^{2}$	*F (df1,df2)*	*p*	$\eta_{p}^{2}$	*F (df1,df2)*	*p*	$\eta_{p}^{2}$
Secure attachment	Experimental	47.658 ± 7.504	51.316 ± 6.921	3.703 (1,150)	0.056	0.024	17.739 (1,150)	<0.001	0.106	4.065 (1,150)	0.046	0.026
	Control	46.816 ± 8.074	48.105 ± 7.183									
Anxious attachment	Experimental	22.289 ± 5.406	22.474 ± 5.247	1.465 (1,150)	0.228	0.010	1.081 (1,150)	0.300	0.007	2.278 (1,150)	0.133	0.015
	Control	21.934 ± 5.478	20.934 ± 5.454									
Avoidant attachment	Experimental	22.013 ± 7.308	22.184 ± 7.417	0.279 (1,150)	0.598	0.002	0.160 (1,150)	0.690	0.001	0.542 (1,150)	0.463	0.004
	Control	22.934 ± 7.371	22.355 ± 6.256									

### Differences in Motivational Responses After Attachment Priming

The final selected (best-fitting) models are the maximal GLMM with diagonal covariance structure for liking and representational response and the maximal GLMM with heterogeneous first-order autoregressive covariance structure for evoked response. The results are summarized in Table 3. There were highly significant main effects of face type (all *ps* < 0.001), which means that participants had obvious preference responses to infant faces. In opposition to hypothesis 1, the main effects of group on the three motivational responses were uniformly non-significant (*ps* = 0.218∼0.990). This means that secure attachment priming did not improve the motivational response for adult and infant faces at the same time. Furthermore, face type × group interactions were significant for representational (*p* = 0.047) and evoked responses (*p* < 0.001) but marginally significant for liking (*p* = 0.092), partially supporting hypothesis 2.

**TABLE 3:** Estimates of fixed and random effects in the final best-fitting models (*n* = 152).

Dependent variables	Fixed effects	Unstandardized β	*SE*	*t*	*p*	95% CI
Liking^a^	Intercept	3.330	0.233	14.294	<0.001	2.862, 3.799
	Face	2.279	0.311	7.336	<0.001	1.65, 2.909
	Group	0.003	0.233	0.012	0.990	−0.455, 0.461
	Face × Group	0.491	0.290	1.694	0.092	−0.082, 1.065
Representational^b^	Intercept	−8.517	1.030	−8.265	<0.001	−10.601, −6.432
	Face	10.682	1.381	7.734	<0.001	7.866, 13.499
	Group	0.427	0.990	0.431	0.667	−1.524, 2.378
	Face × Group	2.488	1.240	2.006	0.047	0.038, 4.939
Evoked^c^	Intercept	−6.656	2.081	−3.198	0.005	−11.016, −2.296
	Face	18.637	3.036	6.138	<0.001	12.325, 24.949
	Group	−2.208	1.781	−1.239	0.218	−5.738, 1.322
	Face × Group	14.537	2.745	5.297	<0.001	9.118, 19.956

**Dependent variables**	**Random effects**	**Estimate**	***SE***	***Wald Z***	***p***	**95% CI**

Liking	Intercept for subject	0.488	0.174	2.799	0.005	0.242, 0.984
	Intercept for slide	0.217	0.087	2.508	0.012	0.100, 0.475
	Slopes for face type	1.373	0.185	7.437	<0.001	1.055, 1.787
Representational	Intercept for subject	10.418	3.291	3.166	0.002	5.610, 19.350
	Intercept for slide	4.535	1.892	2.396	0.017	2.002, 10.275
	Slopes for face type	20.997	3.363	6.243	<0.001	15.34, 28.741
Evoked	Intercept for subject	21.457	11.668	1.839	0.066	7.391, 62.293
	Intercept for slide	16.055	8.330	1.927	0.054	5.808, 44.384
	Slopes for face type	82.057	16.987	4.831	0.000	54.69, 123.119

*^a^Rating scores for each slide.*

*^b^Net keystrokes to seek future exposure to each slide.*

*^c^Net keystrokes to seek current exposure to each slide (prolong the viewing time).*

*Both group and face were dummy coded (1 = experimental group/infant face, 0 = control group/adult face).*

**FIGURE 3:**
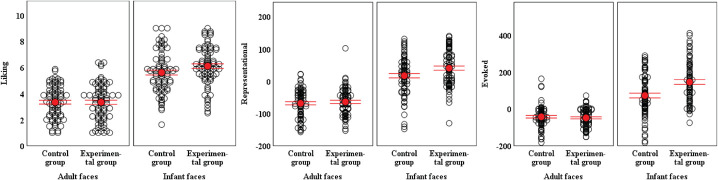
Univariate scatterplots of motivational responses to the different types of faces in each group. The error bars represent ± 1 standard error (red line) of the mean (red point). For the representational and evoked responses, the positive value means positive motivation on average of seeking stimuli again (representational) and prolonging exposure to stimuli (evoked), whereas the negative value means negative motivation to adult or infant faces of avoiding to see stimuli again (representational) and exposure to stimuli (evoked).

The Bonferroni-corrected pairwise comparisons are summarized in Table 4. We can see that the group differences in three motivation indicators for adult faces were uniformly not significant, suggesting that secure attachment priming consistently did not enhance the motivation toward adult faces. In contrast, the experimental group had significantly higher liking ratings and stronger representational and evoked responses for infant faces than the control group (*ps* = 0.0000002∼0.037), indicating that secure attachment priming significantly enhances the approach motivation toward infant faces. Figure 3 was plotted to make the above results more intuitive.

**TABLE 4:** Descriptive statistics and pairwise comparisons (*n* = 152).

Dependent variables	Face type	Group (*M* ± *SD*)^a^		Pairwise comparisons between two groups in each face type			
			
		Experimental	Control	*t*	*df*	*Adj. Sig^b^*	*d*
Liking	Infant	6.100 ± 2.034	5.609 ± 2.137	**2.097**	**291**	**0.037**	**0.340**
	Adult	3.334 ± 1.868	3.339 ± 1.805	0.012	273	0.990	0.002
Representational	Infant	5.082 ± 10.906	2.156 ± 11.827	**2.784**	**305**	**0.006**	**0.452**
	Adult	−7.980 ± 8.870	−8.546 ± 8.567	0.432	239	0.666	0.070
Evoked	Infant	24.261 ± 28.038	12.121 ± 26.095	**5.277**	**436**	** < 0.001**	**0.856**
	Adult	−8.075 ± 14.107	−7.121 ± 14.237	–1.245	111	0.216	–0.202

*^a^The mean and standard deviation are calculated using the score of each slide.*

*^b^The significance level adjusted by the sequential Bonferroni procedure is 0.05. Significant results were highlighted in bold.*

### The Relationship Between Attachment State and Specific Motivational Response to Infant Faces

Since the main effects of face type are significant, we further used the response to adult faces as a baseline to calculate the specific motivational response to infant faces. Then, hierarchical regression was used to investigate whether the state of secure attachment of the two groups could effectively predict their specific motivational responses after attachment priming. As shown in Table 5, after controlling for gender and the states of anxious attachment and avoidant attachment, the state of secure attachment had significant predictive effects on the five specific motivational responses to infant faces.

**TABLE 5:** Summary of hierarchical regression analyses after attachment priming (*n* = 152).

Models		Statistics	Independent variables				*R* ^2^	Δ*R*^2^
			Gender	Anxious attachment	Avoidant attachment	Secure attachment		
SL	Step1	*β*	0.092	0.123	−0.128		0.033	
		*p*	0.257	0.136	0.118		0.168	
	Step2	*β*	0.034	0.055	−0.048	**0.261**	0.090	**0.056**
		*p*	0.672	0.503	0.566	**0.003**	0.008	**0.003**
SR	Step1	*β*	0.092	0.104	−0.101		0.025	
		*p*	0.261	0.207	0.219		0.293	
	Step2	*β*	0.047	0.052	−0.039	**0.202**	0.058	**0.034**
		*p*	0.568	0.537	0.645	**0.023**	0.064	**0.023**
SE	Step1	*β*	0.056	0.157	−0.171		0.048	
		*p*	0.490	0.055	0.037		0.064	
	Step2	*β*	0.009	0.103	−0.106	**0.212**	0.085	**0.037**
		*p*	0.913	0.218	0.209	**0.016**	0.011	**0.016**

*SL, SR, and SE represent the unique scores for liking, representational and evoked responses for the infant faces; regression coefficients reported are standardized; gender was dummy coded (male = 0, female = 1). Significant results were highlighted in bold.*

## Discussion

Consistent with Cheng et al. (2015b), this study confirmed that the state of secure attachment of childless adults could effectively predict their self-reported interest in infants after controlling for gender and states of anxious and avoidant attachment. Second, according to the data obtained before and after attachment priming, the experimental group and the control group were highly homogeneous in attachment orientation, attachment state, and interest in infants before priming. After attachment priming, only the experimental group improved significantly in the state of secure attachment out of the three attachment state indicators, which indicated that attachment priming was effective in the study.

Since secure attachment priming was successful, we then examined the differences between the experimental group and the control group in the motivational responses to infant and adult faces. First, the experimental group scored significantly higher on liking, representational and evoked responses to infant faces than the control group. This proves that with the improvement of the secure attachment state, the motivation to approach infants is strengthened. However, the face type × group interaction was marginally significant for liking. Since the effect sizes were small for *p* values between 0.05 and 0.1 (Olsson-Collentine et al., 2019), current results could not support that the promotion of a state of secure attachment enhances participants’ hedonic experience (liking) for infant faces more than for adult faces. But for representational and evoked response, the interaction effects were significant after considering the random intercepts for both slides and subjects and the random slopes for face type. The results indicated that secure attachment priming amplified the approach motivation to infant more than the approach motivation to adult face.

Notably, the effect sizes of group differences in three motivation to infant ranged from small to large (*d*_*liking*_ = 0.34: small, *d*_*representational*_ = 0.45: medium, and *d*_*evoked*_ = 0.86: large), and a similar trend was shown for the interaction effects. The reason for this phenomenon might be the different task types for the three motivation measures. As the pleasure ratings for infant faces were already significantly higher than those for adult faces, the room for enhancement was relatively small. However, the numbers of key presses within a certain time to seek stimuli or to prolong viewing time have more room for variation. Here, the time available to press keys to seek future exposure (2 s) was shorter than the maximal time available to press keys to prolong viewing time (5 s). Thus, the strongest effect was observed for evoked response. More importantly, given that evoked response was defined as measuring the mixed motivation of liking and wanting, which has been explained in detail in the introduction, we do not think the inconsistent interaction effects reflected that security priming had different roles in different components of motivation. Therefore, we believe that the results of this study basically support the promotion of secure attachment, resulting in an increase in positive motivation for infants.

In addition, we found that there were consistently no differences in the motivational responses to adult faces between the two groups, which was different from what we had expected. According to the results of previous studies (Dan and Raz, 2012; Kafetsios et al., 2014; Zheng et al., 2015; Petrowski et al., 2019; Alaei et al., 2020), adult attachment orientation plays a role in the response to adult faces. Another study demonstrated that changes in individuals’ attachment state can affect their response to adult faces (Tang et al., 2017). However, in this study, the improvement of the secure attachment state did not increase the motivational response to adult faces. In view of this result, we further analyzed the existing literature and found that in these studies (Dan and Raz, 2012; Kafetsios et al., 2014; Zheng et al., 2015; Tang et al., 2017; Petrowski et al., 2019), attachment affected an individual’s response to adult faces because the adult faces used had a variety of expressions; thus, attachment affects an individual’s response to expressive adult faces. In fact, in these studies, as in this study, attachment had no effect on the response to neutral adult faces.

Bowlby (1969/1982) proposed several concepts, including the attachment behavioral system and caregiving behavioral system, based on the behavioral system concept from ethology. Among them, the attachment behavioral system is considered to be activated only when people encounter threats or stressors; it is usually activated only by certain stimuli or situations in which a particular set goal is made salient (Mikulincer and Shaver, 2016). Therefore, based on the above results, because expressive adult faces carry a large amount of emotional information, they can activate the adult attachment behavioral system, which leads to different behavioral reactions among individuals. Due to the lack of emotional value of neutral adult faces, an individual’s response to them is not affected by adult attachment (Dan and Raz, 2012; Kafetsios et al., 2014; Zheng et al., 2015; Tang et al., 2017; Petrowski et al., 2019; Alaei et al., 2020). In fact, this kind of behavior response pattern saves energy and thus represents an evolutionary adaptation.

Moreover, according to the view of the attachment behavioral system of Bowlby (1969/1982), only when the system is deactivated can individuals direct their limited resources to other behavioral systems, such as the caregiving behavioral system. As this study shows, with the improvement of the secure attachment state, the individual’s approach motivation for infant faces (i.e., efforts made to see infants again and to view them longer) is significantly enhanced. This means that the individual’s caregiving behavioral system is activated, but it does not respond to all types of stimuli, such as the attachment behavioral system. The caregiving behavioral system may also make a set goal salient; therefore, it affects only an adult’s response to infant face and has no effect on the response to adult neutral faces, which lack signal value.

Finally, the hierarchical regression analysis of the two groups after attachment priming of this study is consistent with existing findings (Cheng et al., 2015b; Ding et al., 2016). Even after attachment priming, there is a significant linear relationship between the adult secure attachment state and motivational responses, so hypothesis 3 is arguably supported by the results.

In summary, this study has demonstrated for the first time that the promotion of the state of adult secure attachment can effectively enhance approach motivation only to infant faces. This strongly suggests that there is a significant covariant association between adult secure attachment and motivation to approach infants, compensating for the deficiency of previous cross-sectional studies (Cheng et al., 2015b; Ding et al., 2016). This means that in clinical practice, we can enhance the positive reaction of adults to infants by improving the state of adult secure attachment, thereby improving the quality of parenting. Furthermore, the findings contribute to enhancing our understanding of the association between the attachment behavioral system and the caregiving behavioral system. To the best of our knowledge, the current study is the first to prove that the adult caregiving behavioral system, like the attachment behavioral system, makes a particular set goal salient, and it can be activated only by the unique schema features of infant faces and will not produce the same response to all human faces. Whether such a working model is applicable to other behavioral systems has become a question worth exploring.

Although this study provides some useful information, it still has some obvious limitations. First, this study sampled adult college students with no birth experience, and thus, the external validity of the current findings is limited by the fact that adult college students are less likely and less willing, in the short term, to give birth to the next generation. Indeed, given that existing studies have found that interest in infants varies across the life span (Maestripieri and Pelka, 2002; Seifritz et al., 2003; Nishitani et al., 2011), it is necessary to confirm these results in other samples. In addition, the differences between the two groups with regard to the state of secure attachment were achieved through attachment priming. Finally, given that the results were produced under laboratory conditions, whether these findings apply in a naturalistic context will need to be further investigated.

## Data Availability Statement

The raw data supporting the conclusions of this article will be made available by the authors, without undue reservation.

## Ethics Statement

The studies involving human participants were reviewed and approved by The Ethics Committee of Southwest University. The patients/participants provided their written informed consent to participate in this study. Written informed consent was obtained from the individual(s), and minor(s)’ legal guardian/next of kin, for the publication of any potentially identifiable images or data included in this article.

## Author Contributions

FD, YJ, GC, and DZ designed this study and analyzed the data. FD, YJ, and GC drafted the manuscript. All authors contributed to the revision of the first draft and the collection of data.

## Conflict of Interest

The authors declare that the research was conducted in the absence of any commercial or financial relationships that could be construed as a potential conflict of interest.

## Publisher’s Note

All claims expressed in this article are solely those of the authors and do not necessarily represent those of their affiliated organizations, or those of the publisher, the editors and the reviewers. Any product that may be evaluated in this article, or claim that may be made by its manufacturer, is not guaranteed or endorsed by the publisher.
