# Supplementary material for: Secure Attachment Priming Amplifies Approach Motivation for Infant Faces Among Childless Adults
Source: Front Psychol. 2021 Oct 27;12:736379. doi: 10.3389/fpsyg.2021.736379 (PMC8578675; doi:10.3389/fpsyg.2021.736379)
Supplement: Supplementary file 1 [file Table_1.DOCX]

Table S1. Estimates of fixed and random effects in the final best-fitting models (*n* = 152)

| Dependent variables | Fixed Effects | Unstandardized  *β* | *SE* | *t* | *p* | *95% CI* |
| --- | --- | --- | --- | --- | --- | --- |
| Arousal | Intercept | 3.003 | 0.242 | 12.424 | <0.001 | 2.518, 3.489 |
|  | face | 2.268 | 0.314 | 7.233 | <0.001 | 1.632, 2.904 |
|  | group | -0.201 | 0.245 | -0.824 | 0.411 | -0.683, 0.280 |
|  | face×group | 0.714 | 0.287 | 2.486 | 0.014 | 0.147, 1.281 |
| Dominance | Intercept | 3.164 | 0.234 | 13.536 | <0.001 | 2.695, 3.633 |
|  | face | 2.187 | 0.307 | 7.122 | <0.001 | 1.565, 2.809 |
|  | group | 0.051 | 0.239 | 0.213 | 0.831 | -0.419, 0.521 |
|  | face×group | 0.441 | 0.290 | 1.521 | 0.130 | -0.132, 1.014 |
| Dependent variables | Random effects | Estimate | *SE* | *Wald Z* | *p* | *95% CI* |
| Arousal | subjects | 0.746 | 0.197 | 3.783 | <0.001 | 0.444, 1.252 |
|  | slides | 0.228 | 0.091 | 2.494 | 0.013 | 1.565, 2.809 |
|  | face | 1.318 | 0.181 | 7.295 | <0.001 | -0.419, 0.521 |
| Dominance | subjects | 0.616 | 0.186 | 3.304 | 0.001 | 0.340, 1.115 |
|  | slides | 0.209 | 0.084 | 2.483 | 0.013 | 0.095, 0.459 |
|  | face | 1.362 | 0.184 | 7.386 | <0.001 | 1.044, 1.776 |

Note. Both group and face were dummy coded (1=experimental group\adult face, 0=control group\infant face).

Table S2. Descriptive statistics and pairwise comparisons (n=152)

| Dependent variables | Face type | Group (*M± SD*) | | Pairwise comparisons between two groups in each face type | | | |
| --- | --- | --- | --- | --- | --- | --- | --- |
|  |  | Experimental | Control | *t* | *df* | *Adj. Sig^a^* | *d* |
| Arousal | Infant | 5.771±2.301 | 5.876±2.329 | **2.06** | **277** | **0.040** | **0.335** |
|  | Adult | 2.819±1.786 | 2.997±1.783 | -0.83 | 258 | 0.410 | 0.134 |
| Dominance | Infant | 5.837±2.149 | 5.354±2.269 | **2.02** | **287** | **0.044** | **0.328** |
|  | Adult | 3.227±1.869 | 3.166±1.717 | 0.21 | 262 | 0.831 | 0.035 |

^a^ The sequential Bonferroni adjusted significance level is 0.05.

Table S3. Summary of hierarchical regression analyses after attachment priming (*n* = 152)

| Models | | Statistics | Independent Variables | | | | *R^2^* | *△R^2^* |
| --- | --- | --- | --- | --- | --- | --- | --- | --- |
|  |  |  | Gender | Anxious attachment | Avoidant attachment | Secure attachment |  |  |
| SA | Step1 | *β* | 0.117 | 0.079 | -0.107 |  | 0.027 |  |
|  |  | *p* | 0.151 | 0.338 | 0.194 |  | 0.254 |  |
|  | Step2 | *β* | 0.057 | 0.009 | -0.023 | **0.272** | 0.088 | **0.061** |
|  |  | *p* | 0.482 | 0.916 | 0.782 | **0.002** | 0.008 | **0.002** |
| SD | Step1 | *β* | 0.146 | 0.111 | -0.065 |  | 0.033 |  |
|  |  | *p* | 0.074 | 0.177 | 0.429 |  | 0.171 |  |
|  | Step2 | *β* | 0.101 | 0.059 | -0.003 | **0.203** | 0.067 | **0.034** |
|  |  | *p* | 0.221 | 0.483 | 0.976 | **0.022** | 0.036 | **0.022** |

Note*.* SA and SD represent the unique scores for arousal and dominance for the infant faces; regression coefficients reported are standardized; gender was dummy coded (male = 0, female = 1).


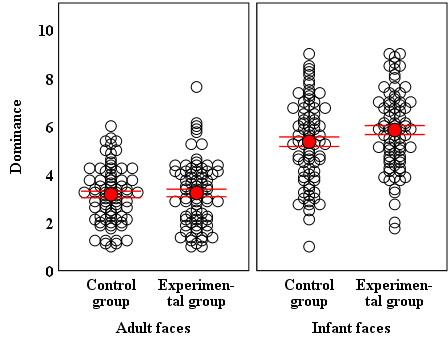

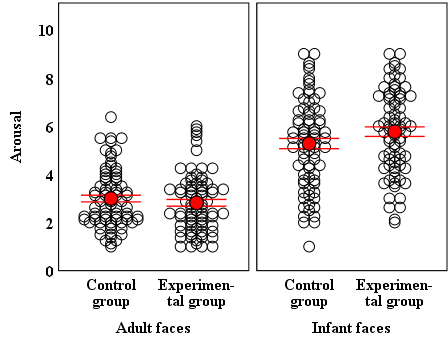


Figure S1. Univariate scatterplots of arousal and dominance ratings for the different types of faces in each group
